# EsigPBP3 Was the Important Pheromone-Binding Protein to Recognize Male Pheromones and Key Eucalyptus Volatiles

**DOI:** 10.3390/ijms25052940

**Published:** 2024-03-03

**Authors:** Hengfei Fu, Guipeng Xiao, Zhende Yang, Ping Hu

**Affiliations:** 1 Guangxi Colleges and Universities Key Laboratory for Cultivation and Utilization of Subtropical Forest Plantation, Guangxi Key Laboratory of Forest Ecology and Conservation, College of Forestry, Guangxi University, Nanning 530004, China; 2109392004@st.gxu.edu.cn (H.F.); dzyang68@126.com (Z.Y.); 2 Biotechnology, Faculty of Science, Autonomous University of Madrid, 28049 Madrid, Spain; guipeng.xiao@estudiante.uam.es

**Keywords:** pheromone-binding proteins, fluorescence competition binding assays, molecular docking, pheromone

## Abstract

Pheromone-binding proteins (PBPs) are specific odorant-binding proteins that can specifically recognize insect pheromones. Through transcriptional analysis of the antennae of adult *Endoclita signifer*, *EsigPBP3* was discovered and identified, and *EsigPBP3* was found to be highly expressed in the antennae of male moths. Based on the binding characteristics and ability of EsigPBP3, we can find the key ligands and binding site to consider as a target to control the key wood bore *E. signifier.* In this study, the fluorescence competitive binding assays (FCBA) showed that EsigPBP3 had a high binding affinity for seven key eucalyptus volatiles. Molecular docking analysis revealed that EsigPBP3 had the strongest binding affinity for the sexual pheromone component, (3E,7E)-4,7,11-trimethyl-1,3,7,10-dodecatetraene. Furthermore, same as the result of FCBA, the EsigPBP3 exhibited high binding affinities to key eucalyptus volatiles, eucalyptol, *α*-terpinene, (E)-beta-ocimene, (−)-*β*-pinene, and (−)-*α*-pinene, and PHE35, MET7, VAL10, PHE38, ILE52, and PHE118 are key sites. In summary, EsigPBP3 exhibits high binding affinity to male pheromones and key volatile compounds and the crucial binding sites PHE35, MET7, VAL10, PHE38, ILE52, and PHE118 can act as targets in the recognition of *E. signifier* pheromones.

## 1. Introduction

Insects are a highly diverse and numerous groups of organisms on Earth. Over time, their population has evolved various biological adaptations to face the complex environment they live in, resulting in the development of sophisticated chemical sensing systems for perceiving a wide range of environmental chemical cues [1,2]. The olfactory system is capable of perceiving and identifying various chemical odor molecules in the environment, including pheromones, plant volatiles, animal scents, and more. This ability guides insects in behaviors such as host location, food selection, mate choice, habitat selection, and evasion of predators [3]. The olfactory system involves the participation of several olfaction-related proteins during the olfactory recognition process, including odorant-binding proteins (OBPs), chemosensory proteins (CSPs), odorant receptors (Ors), gustatory receptors (GRs), ionotropic receptors (Irs), and sensory neuron membrane proteins (SNMPs) [4].

OBPs are a type of small, soluble proteins that play a critical role in the olfactory system of insects, facilitating odor reception. OBPs serve as the first mediators of communication between insects and their external environment [5]. OBPs can transport hydrophobic odor molecules through the aqueous lymph to reach odor receptors, thereby guiding the insect biochemical response to odors. Furthermore, it has been verified that most insect OBPs are capable of binding to host plant volatiles and recognizing pheromones [6]. In recent years, with the rapid development of molecular biology and bioinformatics technologies, an increasing number of OBPs have been successfully identified in various insects. Based on their functions, OBPs can be categorized into three groups: general odorant-binding proteins (GOBPs), pheromone-binding proteins (PBPs), and antennae-specific proteins (ASPs) [7]. Among these, PBPs play a crucial role in the recognition of pheromones. In 1981, Vogt et al. used a sex pheromone labeling method to discover the first odorant-binding protein in the antennae of male moths of the *Antheraea polyphemus* and named it as the pheromone-binding protein (PBP) [8]. So far, multiple PBPs have been identified, primarily in moths of the order Lepidoptera, such as Saturniidae, Sphingidae, and Noctuidae, and they have cloned the genes encoding their *PBP genes* [9].

PBPs contain the typical features of odorant-binding proteins, which are acidic, water-soluble small proteins consisting of 120–160 amino acids, typically around 14–17 kDa, with an isoelectric point of 4–5. Same as OBPs, PBPs exhibit a high degree of protein sequence conservation and possess six conserved cysteine residues, forming three specific disulfide bonds [10]; this structural arrangement stabilizes the protein structure and regulates the binding and release of pheromone molecules. Many studies have indicated that PBPs can bind with pheromones in the lymph of olfactory sensors; for example, three PBP (PxylPBP1, PxylPBP2, and PxylPBP3) of *Plutella xylostella*, all of which exhibited a high affinity for sex pheromones [11]. In addition to using molecular docking, the interaction mechanism between *Bombyx mori* PBPs and sex pheromone components was revealed initially [12]. Moreover, the amino acid residue Trp114 was further identified to be the key site to recognize the major component of *Lobesia botrana* sex pheromone, E7, Z9-12: Ac [13]. It has also been found that different PBPs have varying abilities to bind to sex pheromones. In the FCBA of PBPs of *Lymantria dispar* to the sex pheromone components Z9-14:Ac, Z7-12:Ac, and Z11-16, it was shown that MlorPBP1 had high affinity for all three components, while MlorPBP2 and MlorPBP3 only bound to one component each [14]. The experimental results of the combination of *Grapholitha funebrana* PBPs and sex pheromone ligands show that although all four recombinant GfunPBPs (rGfunPBPs) had binding activity with the tested sex pheromone compounds, their preferred ligands were significantly different [15]. Two types of *SlitPBPs* (*SlitPBP1* and *SlitPBP2*) of *Spodoptera litura* were genetically knocked out using the CRISPR/Cas9 system. GC-EAD analysis of male moths showed that the electrophysiological responses of both were reduced, with male moths lacking *SlitPBP1* exhibiting significantly lower responses than those lacking *SlitPBP2* [16]. The above research shows that some PBPs play a critical role in sex pheromone recognition, driving insect recognition of sex pheromones and generating behavior [17].

In addition to binding to sex pheromones, PBPs can also bind to volatile odor molecules emitted by host plants. For example, EoblPBP2 from *Ectropis obliqua* can bind to various plant odor molecules, including sex pheromone components, suggesting it may be a pheromone-binding protein with a special dual function [18]. CsasPBP1 from *Carposina sasakii* can bind not only to sex pheromone components but also to a wide range of apple volatiles [19]. PBPs can also modulate different courtship behaviors in the communication of sex pheromones. In moths, the specific expression of PBPs can regulate their mating behavior. For most moths, sexual pheromones are detected by proteins that bind to male moth pheromones. However, in *Drosophila melanogaster*, LUSH, as a sex-pheromone-binding protein, is expressed in both male and female insects. The compound cVA can elicit different behaviors in male and female flies, either attracting females or repelling males. This indicates that sex-pheromone-binding proteins can modulate diverse mating behaviors in insects [20,21].

The ghost moth, *E. signifer* Walker (Lepidoptera, Hepialidae), is a wood-boring pest recently discovered in the Guangdong and Guangxi regions, which poses a threat to eucalyptus trees [22]. Its primary harm is caused by the larval stage, as they bore into the tree trunks, affecting tree growth or leading to wind breakage, resulting in significant economic and ecological losses [23]. Due to the complex life history of the *E. signifer* and its characteristic of drilling and feeding on columns, the harm is concealed, and it has become a disaster when discovered [24]. Conventional prevention and control methods are difficult to be effective. Therefore, using sex pheromones or plant volatiles to detect and control its adults is an ideal control method. When observing the mating behavior of adult *E. signifer*, it was found that male wasps attract females from a distance, while at close range, males chase females in two different mating behaviors. These two behaviors reflect the existence of a special sex pheromone communication process in *E. signifer* [25]. Moreover, the high concentration of *E. signifer* hair-pencils extract can induce antennal potential responses in male and female moths, so it is speculated that *E. signifer* may rely on male moth sex pheromones for olfactory communication. Observations were made on the mating and oviposition behavior of adult *E. signifer*. The adults of *E. signifer* exhibit a relatively obvious selective behavior toward different host plants, and they tend to prefer mating and ovipositing on *Eucalyptus grandis* × *Eucalyptus urophylla* in the forest. Similar selection behavior has also been found in other Lepidoptera insects [26]. Anderson [27] found that female adults chose to oviposit on host plants that their larvae fed on. In addition, male adults are more easily attracted by female sex pheromones combined with the volatile substances of host plants that they experienced during their larval stage. Therefore, it is speculated that adult *E. signifer* affect their mating and oviposition behavior by recognizing the volatile substances emitted by eucalyptus leaves.

In addition, the male pheromone of *E. signifer* was identified as (3E,7E)-4,7,11-trimethyl-1,3,7,10-dodecatetraene, and we found that 23 key volatile compounds were extracted from *E. grandis × E. urophylla* leaves. Then, 12 plant volatiles including camphene, benzene, 1,2-diethyl-, eucalyptol, (−)-*α*-pinene, (−)-*β*-pinene, *α*-phellandrene, n-butyl ether, 4-ethylacetophenone, 2-phenyl-2-propanol, d-limonene, butyl acrylate, and 1,3,5-trimethyl-benzen were screened with high content or caused obvious antennal responses of GC-EAD, which were the key volatiles to *E. signifer*. Three PBPs were identified through transcriptomic analysis of the adult male antennae in the *E. signifer*. Notably, *EsigPBP3* exhibited higher expression levels in the male antennae. It is hypothesized that *EsigPBP3* may be one of the key PBPs involved in binding critical volatile compounds and male pheromones. Therefore, in this study, through FCBA and molecular docking between EsigPBP3 and key volatile compounds [28], the binding characteristics of EsigPBP3 and its roles in the odor recognition of *E. signifer* were further explored. We can find the key ligands and binding sites to consider as targets to control *E. signifier*.

## 2. Results

### 2.1. Sequence Characteristics of EsigPBP3

**Figure 1:**
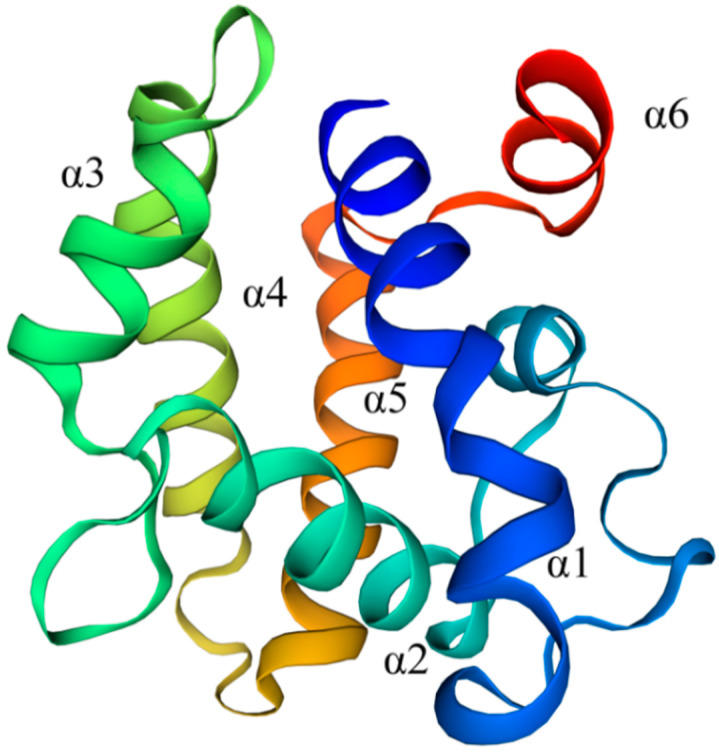
The 3D model of EsigPBP3 of *E. signifier*. Contains six alpha helices: Lys4-Ser25 (*α*1), Arg46-Lys57 (*α*2), His70-Lys78 (*α*3), Glu84-Lys100 (*α*4), Asp107-Glu124 (*α*5), and Val131-Ile138 (*α*6).

**Figure 2:**
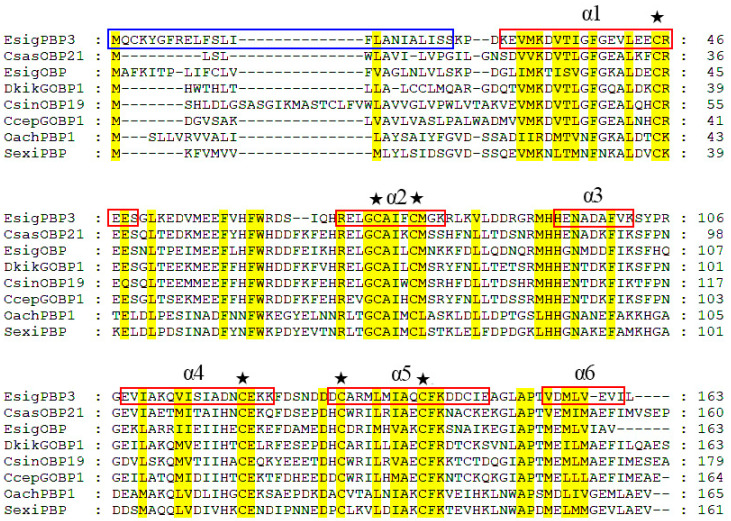
Characterization of the amino acid sequence of *EsigPBP3*. Sequence source: CsasOBP21: *C. sasakii* (AYD42196.1); EsigOBP: *E. signifer* (UVX20220.1); DkikGOBP1: *Dendrolimus kikuchii* (AGJ83357.1); CsinOBP19: *Conopomorpha sinensis* (QGN03649.1); CcepGOBP1: *Corcyra cephalonica* (UDM59724.1); OachPBP1: Orthaga achatina (AEZ52490.1); SexiPBP: *Synanthedon exitiosa* (AAF06123.1). The blue box shows the position of the signal peptide; the red box shows the α-helices position. ★ represents the six conserved cysteines.

The ORF of *EsigPBP3* is 492 bp in length, encoding 163 amino acids, with an N-terminal signal peptide consisting of 24 amino acids. The three-dimensional structure of EsigPBP3 was constructed using the SWISS MODEL (Figure 1). The QMEAN total score is 0.78, with a 51.09% alignment consistency to *B. mori* GOBP2 (PDB ID: 2wc6.1), sharing 47% similarity and covering 99% of the structure. This structure contains six *α*-helices: Lys4-Ser25 (*α*1), Arg46-Lys57 (*α*2), His70-Lys78 (*α*3), Glu84-Lys100 (*α*4), Asp107-Glu124 (*α*5), and Val131-Ile138 (*α*6) (Figure 1). It possesses six conserved cysteine residues, and it was predicted that these six cysteine residues form three pairs of disulfide bonds (Figure 2).

### 2.2. Bacterial Expression and Purification of EsigPBP3

**Figure 3:**
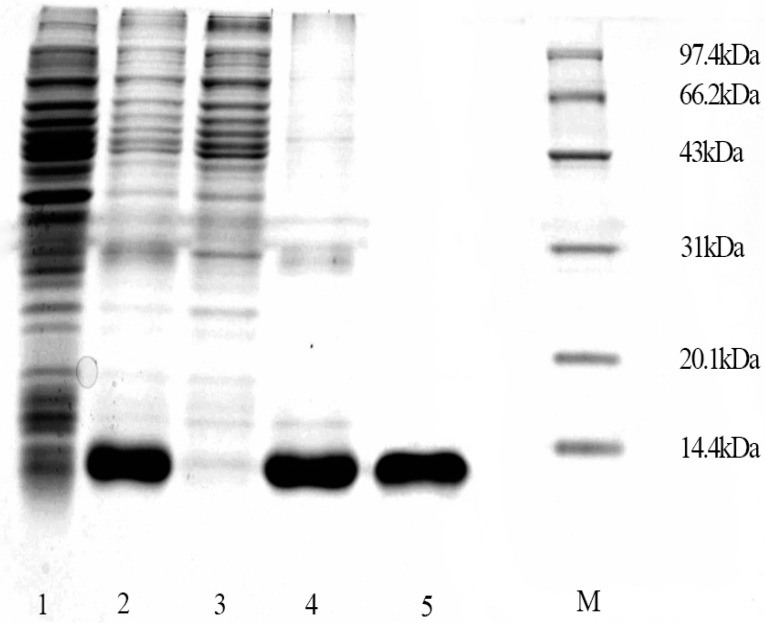
Expression and purification of EsigPBP3. Lane 1 represents uninduced pET + PBP3 without IPTG induction; Lane 2 represents induced pET + PBP3; Lane 3 represents the supernatant after ultrasonication of pET + PBP3; Lane 4 represents the precipitate after ultrasonication of pET + PBP3; Lane 5 represents the purified refolded PBP3 protein.

EsigPBP3 was successfully expressed in the form of inclusion bodies, as the recombinant protein mainly resided in the deposits after cell lysis and centrifugation. Affinity chromatography purification of the EsigPBP3 protein indicated that 50 mM imidazole could be used for protein purification, while 250 mM imidazole was suitable for eluting the target protein. The purified protein was confirmed on an SDS-PAGE gel, showing specific protein bands at approximately 14 kDa (Figure 3). Finally, the protein underwent denaturation and renaturation processes, resulting in a soluble purified protein.

### 2.3. Binding Affinity of EsigPBP3 to Functional Compounds

**Figure 4:**
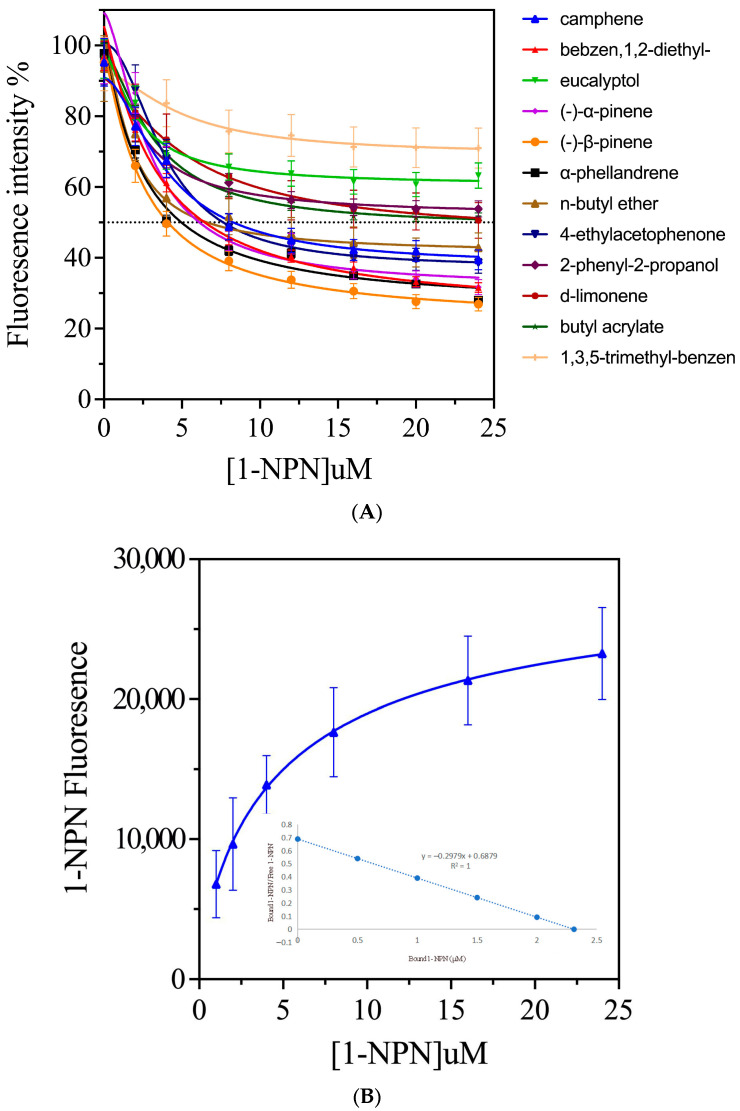
Fluorescence competitive binding curves of PBP3 and the volatile matter. (**A**) The binding curves of EsigPBP3 and 12 compounds; The position of the dashed line represents the location where the fluorescence intensity of the olfactory protein-NPN complex decreases by half. The intersection with the curve indicates the concentration of ligand odor molecules when the fluorescence intensity of the complex decreases by half. (**B**) Binding curve and Scatchard analysis of EsigPBP3 with 1-NPN.

To determine whether EsigPBP3 specifically binds to plant volatiles with GC-EAD activity, we assessed the binding affinity of the recombinant protein EsigPBP3 to these volatiles using a fluorescence competitive binding assay (FCBA). According to the binding curve and Scatchard plot, EsigPBP3 exhibited high affinity for the reporter ligand 1-NPN with a dissociation constant (K_d_) of 3.357 µM (Figure 4B). Competitive fluorescence binding curves demonstrated that all evaluated ligands reduced the relative fluorescence intensity of the [EsigPBP3/1-NPN] mixture (Figure 4A). The binding affinity (K_i_ values) ranged from 3.200 µM to 31.253 µM. Furthermore, (−)-*β*-pinene exhibited the highest affinity at 3.200 µM, followed by *α*-phellandrene at 3.569 µM, benzene, 1,2-diethyl- at 4.829 µM, (−)-*α*-pinene at 5.272 µM, and 4-ethylacetophenone at 5.547 µM, all showing strong binding to EsigPBP3. Camphene had an affinity of 6.611 µM and n-butyl ether at 6.984 µM (Table 1). Therefore, the results indicate that EsigPBP3 is a functional protein that enables *E. signifer* to recognize eucalyptus volatiles, with the highest affinity observed for (−)-*β*-pinene, *α*-phellandrene, benzene, 1,2-diethyl-, and (−)-*α*-pinene.

### 2.4. Evaluation of the EsigPBP3 Model and Molecular Docking

**Figure 5:**
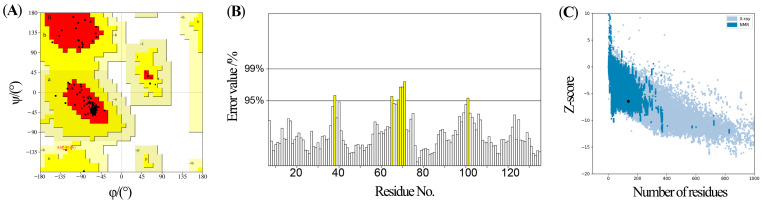
Model evaluation of EsigPBP3 of *E. signifier*. (**A**) The Ramachandran plot of EsigPBP3. The red A, B, and L regions are the best regions of the protein residues. The bright yellow a, b, l, p regions are the appropriate regions of the protein residues. The soil yellow ~a, ~b, ~l, ~p regions are the barely permitted and irrational area for protein residues. (**B**) The error value from the ERRAT calculation of the modeled *E. signifier* EsigPBP3 structure. White color indicates the error value < 95%; yellow color indicates 95% ≤ the error value < 99%. (**C**) The ProSA-web z-score plot of *E. signifier* EsigPBP3. The blue dots show the structure of natural proteins as measured by NMR. The gray dots represent the natural protein structures determined by X-ray crystal diffraction. The black dots represent the EsigPBP3 protein.

Using the homology modeling approach, a three-dimensional model of EsigPBP3 was constructed, with the template derived from the *B. mori* GOBP2 (PDB ID: 2wc6.1). The evaluation results were shown through a Ramachandran plot generated by the Procheck program (Figure 5A). If over 90% of the amino acid residues are located in the most favored regions, it indicates a high-quality model. Analysis of the conformational plot revealed that 94.4% of the amino acid residues in the EsigPBP3 protein model are in the most favored regions, 4.8% are in the allowed regions, and 0.8% are in the generously allowed regions. The quality of the EsigPBP3 protein model structure was further analyzed using the ERRAT program, resulting in a score of 94.57% (Figure 5B). Generally, when the reasonable region exceeds 50%, it suggests a high degree of rationality in the non-bonded interactions between model atoms and the carbon skeleton’s structure. Global model quality scores (GMQE) from ModFOLD8 with scores greater than 0.4 typically indicate a more complete and reliable model, while a *p*-value less than 0.001 suggests a high level of confidence in the model. The quality score for the EsigPBP3 model was 0.6971, and the *p*-value was 5.276 × 10^−6^. Furthermore, the Z-score plot generated by the PROSA program (Figure 5C) was used to evaluate the model. The Z-score was found to be −6.43, and the EsigPBP3 model structure (represented by black dots) was within the region of natural protein structures (represented by blue dots), indicating the reasonableness of the model structure.

The binding energy of the EsigPBP3 protein with volatile compounds was simulated using the AutoDock tools (ver 1.5.6) software, where a smaller numerical value indicates a stronger binding affinity [29]. A total of twenty-three plant volatile molecules and one insect pheromone, farnesene electronic rearrangement isomer, were docked (Table 2). The binding energies of EsigPBP3 with ligands ranged from −3.14 kcal/mol to −5.98 kcal/mol. Among them, the insect pheromone (3E,7E)-4,7,11-trimethyl-1,3,7,10-dodecatetraene exhibited the lowest binding energy with EsigPBP3 at −5.98 kcal/mol. EsigPBP3 exhibited strong binding affinity with (3E,7E)-4,7,11-trimethyl-1,3,7,10-dodecatetraene. Six compounds with binding energies lower than −5.20 kcal/mol were identified. Apart from (3E,7E)-4,7,11-trimethyl-1,3,7,10-dodecatetraene, the next most favorable binding energies were observed for eucalyptol (CAS: 470-82-6) at −5.37 kcal/mol, *α*-phellandrene (CAS: 99-83-2) at −5.29 kcal/mol, (E)-beta-ocimene (CAS: 13877-91-3) at −5.26 kcal/mol, (−)-*β*-pinene (CAS: 18172-67-3) at −5.16 kcal/mol, and (−)-*α*-pinene (CAS: 7785-70-8) at −5.20 kcal/mol.

### 2.5. Molecular Docking Binding Site

**Figure 6:**
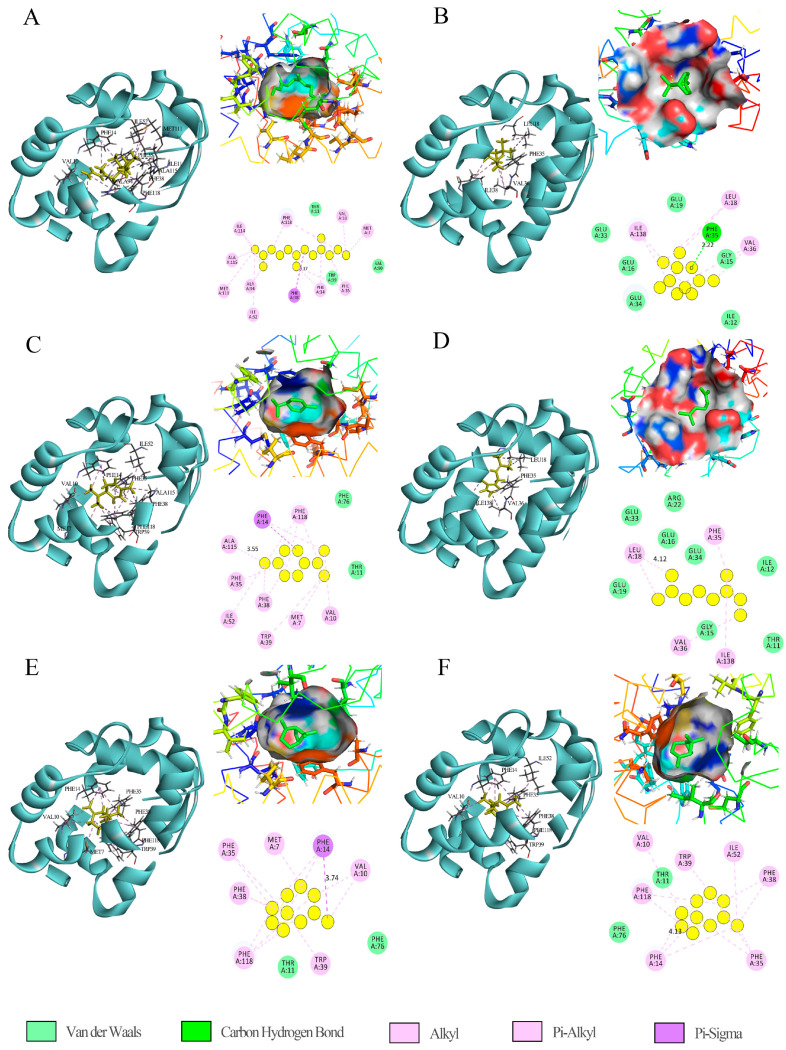
Binding mode diagram of EsigPBP3 and ligand and 2D and 3D schematic diagram.In the 3D schematic diagram, the green rod-like structure inside the binding pocket represents the volatile ligand, while the other colored rod-like structures outside the binding pocket symbolize the amino acid residues bound to the ligand. In the 2D diagram, the yellow circles represent the volatile ligand (**A**) The binding mode of (3E,7E)-4,7,11-trimethyl-1,3,7,10-dodecatetraene and EsigPBP3; (**B**) The binding mode of eucalyptol and EsigPBP3; (**C**) The binding mode of *α*-phellandrene and EsigPBP3; (**D**) The binding mode of (E)-beta-ocimene and EsigPBP3; (**E**) The binding mode of (−)-*β*-pinene and EsigPBP3; (**F**) The binding mode of (−)-*α*-pinene and EsigPBP3.

By analyzing the binding mode of (3E,7E)-4,7,11-trimethyl-1,3,7,10-dodecatetraene and EsigPBP3 (Figure 6A), it was found that the ligand was in the binding cavity formed by the *α*-helix of EsigPBP3. The amino acid residues MET7, VAL10, PHE35, PHE38, ILE52, ALA94, MET111, ILE114, ALA115, and PHE118 were involved in the mutual interaction between the two entities. Specifically, PHE38 formed a σ-π conjugated system with the ligand molecule, representing an important intermolecular interaction force. However, hydrophobic interactions remain crucial for the binding of the ligand to EsigPBP3, with MET7, VAL10, PHE35, PHE38, ILE52, ALA94, MET111, ILE114, ALA115, and PHE118 being key amino acid residues that interact with (3E,7E)-4,7,11-trimethyl-1,3,7,10-dodecatetraene.

The binding mode of eucalyptol and EsigPBP3 was analyzed (Figure 6B), revealing that hydrogen bonds and hydrophobic interactions were the key interactions between eucalyptol and EsigPBP3. The amino acid residues LEU18, PHE35, VAL36 and ILE138 were involved in their interaction. Notably, PHE35 interacted with the ligand through a hydrogen bond with a distance of 2.22 Å, while the remaining interactions were hydrophobic in nature. The predicted amino acid residues LEU18, PHE35, VAL36, and ILE138 were critical binding sites involved in specific interactions with eucalyptol. Among these, PHE35 played a more significant role.

By analyzing the binding mode of *α*-phellandrene with EsigPBP3 (Figure 6C), it was found that there was no hydrogen bonding between them. Hydrophobic interactions and van der Waals forces were the critical interactions between EsigPBP3 and *α*-phellandrene. The amino acid residues MET7, VAL10, PHE14, PHE35, PHE38, TRP39, ILE52, ALA115, and PHE118 were involved in the interaction between the two. MET7, VAL10, PHE14, PHE35, PHE38, TRP39, ILE52, ALA115, and PHE118 were predicted to be the key binding sites for the specific binding of *α*-phellandrene.

The binding mode of (E)-beta-ocimene and EsigPBP3 was analyzed (Figure 6D), revealing that there were no hydrogen bonds formed between them. They primarily interacted through intermolecular forces. Multiple hydrophobic interactions were observed between (E)-beta-ocimene and EsigPBP3, and the crucial amino acid residues involved in binding were LEU18, PHE35, VAL36, and ILE138.

The binding mode of (−)-*β*-pinene with EsigPBP3 was revealed through analysis (Figure 6E). (−)-*β*-pinene was located within the binding cavity formed by the α-helix of EsigPBP3, with hydrophobic interactions being the critical interaction between (−)-*β*-pinene and EsigPBP3. The amino acid residues MET7, VAL10, PHE14, PHE35, PHE38, TRP39, and PHE118 were involved in the interaction between the two, all of which are hydrophobic interactions. Among them, PHE14 had the closest distance to the ligand (3.74 Å), predicting MET7, VAL10, PHE14, PHE35, PHE38, TRP39, and PHE118 as the keys binding sites for the specific binding of (−)-*β*-pinene.

The binding mode analysis of (−)-*α*-pinene with EsigPBP3 (Figure 6F) revealed that hydrophobic interactions were crucial for the interaction between EsigPBP3 and (−)-*α*-pinene. The amino acid residues VAL10, PHE14, PHE35, PHE38, TRP39, ILE52, and PHE118 participated in the interaction between these two, and there were multiple hydrophobic interactions and no hydrogen bonds between EsigPBP3 and (−)-*α*-pinene. It was predicted that VAL10, PHE14, PHE35, PHE38, TRP39, and ILE52 were the key binding sites for specific binding to (−)-*α*-pinene.

## 3. Discussion

The FCBA results showed that EsigPBP3 has a high affinity for seven GC-EAD active ligands, namely, camphene, 1,2-diethylbenzene, (−)-*α*-pinene, (−)-*β*-pinene, *α*-phellandrene, dibutyl ether, and 4-ethylacetophenone. The molecular docking binding energy results indicate that EsigPBP3 has the strongest binding affinity with insect pheromone components, followed by eucalyptol, *α*-phellandrene, (E)-beta-ocimene, (−)-*β*-pinene, and (−)-*α*-pinene. All the result showed that EsigPBP3 was one of the important PBPs to recognize male pheromones and key volatiles in *E. signifer*.

PBPs, as the primary proteins involved in insect mating behavior, were initially believed to be predominantly present in the antennae of male insects. However, with the advancement of molecular techniques, it has been discovered that PBPs are also found in the antennae of female moths in the Lepidoptera order. For instance, in the case of the *C. punctiferalis*, both *CpunPBP3* and *CpunPBP1* exhibit comparable expression levels in the antennae of both male and female moths [30]. *PBPs* are also expressed at different developmental stages and in different organs with variations. In the *Lymantria dispar*, *LdisPBP1* and *LdisPBP2* start expressing even before pupal eclosion, peaking at 1 day before eclosion and persisting through the adult stage [31]. In the *Helicoverpa assulta*, *HassPBP 2* begins its expression in the late pupal stage and continues into the mid-adult stage, with expression detected in both male and female antennae, albeit higher in males [32]. In the *P. xylostella*, three *PBPs* are expressed not only in the antennae of both male and female moths but also in small quantities in the female reproductive organs and male legs [11]. The expression of *PBPs* in different parts of the body also indicates the importance of *PBPs* in insects.

The primary function of PBPs is the specific recognition and responsiveness to sex pheromones, which are typically composed of mixtures of 2–3 compounds produced and released by the female moth pheromone gland [33]. Male moths primarily use specialized sensilla with long hair-like structures on their antennae to detect sex pheromones. Within these sensilla, odorant receptor neurons (ORNs) detect specific pheromone molecules through pheromone receptors expressed on their dendrites [34]. The main role of PBPs is to bind environmental sex pheromones and transport them to the pheromone receptors, thereby triggering the male moth’s search behavior for the source of the pheromone release [1]. The first OBP discovered in the antennae of *A. polyphemus* was believed to be a PBP because of its specific binding affinity to the major component of the sex pheromone, E6, Z11-hexadecenoic acid ester [8]. *Grapholita molesta* GmolPBP2 specifically binds to the two major sex pheromone components of this species, Z8-12: Ac and E8-12: Ac [35]. Similarly, *Orthaga achatina* OachPBP1 exhibits a high binding affinity to all three putative sex pheromones of the species and ten known pheromone analogs [36]. These results are primarily based on in vitro experiments, and in vivo PBP behavioral assays corroborate these findings. Similarly, after RNAi interference of both *HarmPBP1* and *HarmPBP2* in the male *Helicoverpa armigera*, the EAG responses to sex pheromones in the antennae significantly decreased, and the responses were lower than those when interfering with a single gene [37]. The molecular docking results in this study indicate that EsigPBP3 has the highest binding affinity with the insect sex pheromone component (3E,7E)-4,7,11-trimethyl-1,3,7,10-dodecatetraene [28]. This suggests that EsigPBP3 can specifically bind to insect sex pheromone compounds, a function similar to that of most sex-pheromone-binding proteins. Male adults are more attracted to the host plant volatiles experienced during their larval stage when these volatiles are associated with female sex pheromones [27]. It is hypothesized that EsigPBP3 may assist the *E. signifer* in recognizing sex pheromones and plant volatiles in complex odor environments, thereby contributing to the mating and reproductive behavior of the moth.

PBPs, in addition to recognizing sex pheromones, are also involved in the identification of plant volatiles [38,39]. For example, AcorOBP1 from *Anomala corpulenta* can not only bind to the sex pheromone 5-tetradecyl-2-one, but can also bind to host plant volatiles such as Methyl Salicylate and 1-Decanol [40]. In the study of the fluorescence competitive binding and molecular docking of EsigPBP3 and key eucalyptus leaf volatiles, the fluorescence competitive binding results showed that (−)-*β*-pinene had the highest affinity with PBP3, followed by *α*-phellandrene and benzene, 1,2-diethyl-. Combining energy results show that, apart from the sex pheromone component, the binding ability of eucalyptol and PBP3 is the strongest, followed by *α*-phellandrene. Therefore, it is inferred that (−)-*β*-pinene, *α*-phellandrene, and eucalyptol are key ligand molecules that bind to EsigPBP3. (−)-*β*-pinene is a key volatile compound for the specific recognition of eucalyptus. In the behavioral response of fifth instar *E. signifer* larvae to volatiles, only (−)-*β*-pinene exhibits a clear selection behavior. At the same time, (−)-*β*-pinene has the highest affinity with the sex pheromone protein PBP3, indicating that (−)-*β*-pinene is the key ligand molecule that binds to EsigPBP3. According to the results of the GC-EAD measurements, both third and fifth instar *E. signifer* larvae exhibited antennal responses to *α*-phellandrene, indicating that the larvae can specifically recognize *α*-phellandrene. The FCBA and molecular docking results indicate that EsigPBP3 has a high level of binding affinity to *α*-phellandrene, indicating that *α*-phellandrene is a key volatile compound that affects the olfactory recognition of *E. signifer*. Eucalyptol is one of the strongest ligands for binding to EsigPBP3. The GC-MS content measurement shows that eucalyptol has a higher content on eucalyptus leaves and is one of the main components of eucalyptus leaf volatiles. According to the results of GC-EAD, adult *E. signifer* exhibited electrophysiological responses to eucalyptol, indicating their ability to recognize eucalyptol [41]. EsigPBP3 showed a strong binding affinity to eucalyptol, suggesting that EsigPBP3 is a key recognition gene for this compound. Based on the above analysis, EsigPBP3 can specifically recognize key volatile compounds in eucalyptus leaves such as (−)-*β*-pinene, *α*-phellandrene, and eucalyptol, thereby affecting the oviposition behavior of female *E. signifer*. EsigPBP3 is one of the key proteins involved in adult insect olfaction, and it exhibits functional diversity.

Through analysis of the binding sites and binding pockets of EsigPBP3, it was observed that the key binding site for the sex pheromone component (3E,7E)-4,7,11-trimethyl-1,3,7,10-dodecatetraene with EsigPBP3 contains the highest number of amino acid residues, specifically ten. This suggests that the amino acid residues involved in binding are directly proportional to the binding affinity. The most crucial amino acid residue in the binding site between eucalyptol and EsigPBP3 is PHE35, where a short but strong hydrogen bond is formed between the two. Additionally, when examining the binding sites of the sex pheromone component (3E,7E)-4,7,11-trimethyl-1,3,7,10-dodecatriene with five different plant volatiles, it was found that the most important amino acid residues include PHE35, which participates in the binding of all six compounds. Other significant residues involved in multiple volatile bindings are MET7, VAL10, PHE38, ILE52, and PHE118. We found that EsigPBP3 has two binding pockets, and the ligands with higher binding affinities mostly bind between the six α-helices forming the binding pockets at the center of the EsigPBP3 protein. It is speculated that the centrally located binding pocket is the primary binding region with stronger binding capabilities. Similar conformations were also discovered in *Sirex noctilio* SnocOBP6, which likewise has two binding pockets corresponding to its two ligands, z-7-heptanol and 3-carene [42]. The difference is that the two binding pockets in SnocOBP6 almost overlap, whereas EsigPBP3’s binding pockets are independent. In the future, site-specific mutations of EsigPBP3 are needed to validate this hypothesis via in vitro and/or in vivo studies. Ligands interact with the protein in the binding pockets primarily through hydrophobic interactions and hydrogen bonding, along with complementary key residues. Studying these binding pockets and binding sites will provide a theoretical basis for further research and the development of targeted attractants.

## 4. Materials and Methods

### 4.1. Cloning and Sequencing

Total RNA of the male antennal cDNA of *E. signifer* was prepared using TRIzol Reagent (DNA isolation) (Thermo Fisher, Waltham, MA, USA) according to the instructions of the RNeasy Plus Mini Kit (Qiagen, Hilden, Germany). The complementary DNA (cDNA) was synthesized using the EasyScript One-Step gDNA Removal and cDNA Synthesis SuperMix (TransGen, Beijing, China). PCR forward primers (5′-AAGCCGGATAAGGAAGTAATGAAG-3′) and reverse primers (5′-TAAAGGATGACTTCGACCAGCATGT-3′) were designed based on the EsigPBP3 sequence (GenBank Accession AGJ83357.1).

Using the male antennal cDNA of the *E. signifer* as a template, *EsigPBP3* was amplified through PCR (OSE-GP-03, TianGen, Beijing, China). The product was ligated onto the pEASY-T Easy Vector (TransGen, Beijing, China) to construct a recombinant plasmid, which was then transformed into *Escherichia coli* DH5α-competent cells and plated on LB agar medium (1 µL ampicillin: 1 mL LB). Plasmids were extracted using the Plasmid Extraction Mini Kit (Solarbio, Beijing, China). Positive clones were screened and sequenced (Qingke, Beijing, China). The DNA sequence was deposited into GenBank with the accession number AGJ83357.1.

### 4.2. Sequence and Structure Analysis

The Open Reading Frame Finder (ORF Finder) (https://www.ncbi.nlm.nih.gov/orffinder/ (accessed on 28 May 2023)) online website was used to analyze the Open Reading Frame (ORF) of the larva unigene. Using the SignalP-4.1 online tool (https://services.healthtech.dtu.dk/services/SignalP-4.1/ (accessed on 28 May 2023)), we predicted the signal peptide of the *E. signifer* EsigPBP3 amino acid sequence and used the sequence without the signal peptide for subsequent analysis [43]. The ClustalW program in the MEGA (version 5.0) software was used to compare homology between sequences according to the default parameters.

The gene sequence of removing the signal peptide was translated into an amino acid sequence and uploaded to the SWISS-MODEL online software on the ExPASy server (https://swissmodel.expasy.org/interactive (accessed on 29 May 2023)) to search for template proteins in the protein database PDB that match it. First, we looked for homologous proteins with high-resolution 3D structures and selected those with amino acid sequence similarity greater than 30% as templates for homology modeling [44]. Then, we used this template to construct the three-dimensional structure of the EsigPBP3 protein and optimized the generated structure using Modeller (ver 9.14) [45].

The Procheck program available on the UCLA website (http://services.mbi.ucla.edu/ (accessed on 4 June 2023)) was used to evaluate the stereochemical characteristics of protein models based on the distribution of amino acids [46]. The ERRAT program was employed to assess the overall performance of the template by comparing non-bonded interactions between different atoms in the three-dimensional structure and differences from high-resolution crystal structures [47]. Model quality was evaluated by comparing the distances between amino acid residues and their equivalent residues in native protein structures using the ModFOLD 8 online software (https://www.reading.ac.uk/bioinf/ModFOLD/ (accessed on 5 June 2023)) to predict the similarity between the model and the native protein structure [48].

### 4.3. Expression and Purification of EsigPBP3

Based on the sequence of *EsigPBP3*, primers with restriction enzyme recognition sites were designed for enzymatic cleavage. The forward primer 5′-GAATTCAAGCCGGATAAGGAAGTAATGAAG-3′ with the *Eco*RI digestion site, and the reverse primer 5′-AAGCTTTAAAGGATGACTTCGACCAGCATGT-3′ with the *Hind*III digestion site were created. Using a cloning vector plasmid as a template, PCR was performed using the 2× TransTaq High Fidelity (HiFi) PCR SuperMix I (TransGen, Beijing, China), and after completion, the products were analyzed using 1% agarose gel electrophoresis. The recovered products were then ligated to the pEASY-Blunt Simple vector to construct the PBP3-pEASY-Blunt Simple plasmid, which was subsequently transformed into Trans1-T1-phage-resistant chemically competent cells for propagation and sequencing. The sequencing results matched the target fragment sequence. Plasmid extraction was performed, followed by double digestion using EcoRI and HindIII. The resulting target fragment was ligated to the expression vector pET30a(Novagen, Madison, WI, USA) using T4 DNA ligase (New England Biolabs, Ipswich, MA, USA), with the ligation carried out overnight at 16 °C. The plasmid containing the correctly inserted fragment (pET30a-EsigPBP3) was then transformed into *E. coli* BL21-competent cells. A single clone was selected and cultured overnight in 50 mL of medium. The following day, the 50 mL culture was transferred to 200 mL of kanamycin-resistant medium and grown with agitation until it reached the logarithmic growth phase (OD_600_ = 0.6). IPTG (Biotopped, Beijing, China) was added to a final concentration of 1 mM and the culture was shaken for 8 h to induce the expression of EsigPBP3. The bacterial cells were collected, washed 2–3 times with 0.01 M PBS, and resuspended in an appropriate volume of PBS. The suspended bacterial cells were sonicated on ice for 12 min using an ultrasonic disruptor (SCIENTZ-IID, Scientz, Ningbo, China) then centrifuged at 4 °C and 12,000 rpm for 15 min to completely separate the supernatant and bacterial cell fragments. The inclusion bodies were collected, first washed with 2 M urea, then centrifuged at 4 °C and 12,000 rpm for 15 min to collect the precipitate, and dissolved in 8 M urea at pH 8.0. They were washed with an equilibration buffer (0.5 M NaCl, 8 M urea, 20 mM Tris-HCl pH 8.0, 10% glycerol), and then eluted with a buffer containing 10 M to 250 M imidazole in a 20 mM Tris-HCl buffer (pH 8.0) with 8 M urea. Finally, the protein was refolded using a refolding buffer containing 0.5 M to 6 M urea. The protein was concentrated using an Amicon Ultra concentrator (Millipore, Billerica, MA, USA) with a cutoff of 3 kDa. Purity and concentration were determined using a 15% SDS-PAGE method and the Bradford assay, respectively.

### 4.4. Fluorescence Competition Binding Assay

Fluorescence competition assays were conducted using 1-NPN (4-nitrophenyl 2,3-naphthalenedicarboxylate) as a selective fluorescence probe to measure the affinity of ligands for binding to the recombinant EsigPBP3 [49]. These assays were performed on a multi-scan spectral device, SpectraMax i3 (Thermo Scientific, Wilmington, DE, USA), with an excitation wavelength of 337 nm. The emission wavelength range was set from 380 nm to 520 nm. After the fluorescence intensity stabilized, scans were performed and the emission was recorded (both excitation and emission slit widths were set to 10 nm). A final concentration of 2 μM EsigPBP3 solution was prepared in 20 mM Tris-HCl buffer (pH 7.4), and the ligands were dissolved in chromatographically pure methanol as 1 mM stock solutions. The affinity of EsigPBP3 for the labeled probe was determined by adding aliquots of the 1-NPN stock solution to give final concentrations of 2–24 µM. The compound was added proportionally to a mixture with a final concentration of 2 µM EsigPBP3 protein and 2 µM 1-NPN. The ability of ligands to bind to the EsigPBP3 protein was monitored by the decrease in fluorescence intensity. The final concentrations of the twelve ligands ranged from 2 µM to 24 µM, and each assay was repeated six times. The dissociation constant (K_i_) for the binding affinity of each chemical compound to EsigPBP3 was determined based on the maximum half-maximal inhibitory concentration (IC_50_) and the dissociation constant (K_d_) was calculated using the Hill curve fitting equation, θ = [L]^n^/(K_d_ + [L]^n^), where θ represents the score for occupied binding sites where ligands can bind to the active site of the receptor protein at the occupied binding site; [L] denotes the concentration of free (unbound) ligands; K_d_ corresponds to the dissociation equilibrium constant for ligand dissociation; (n) is the Hill coefficient, describing cooperativity (or other biochemical properties, depending on the context of using the Hill equation). Then, we used the formula K_i_ = [IC_50_]/(1 + [1-NPN]/K_1-NPN_), where [1-NPN] is the concentration of 1-NPN, and K_1-NPN_ is the dissociation constant of EsigPBP3/1-NPN [50,51].

### 4.5. Molecular Docking

Based on the CAS numbers of alternative docking ligands, 3D models of the ligand molecules were obtained from the PubChem database on NCBI (https://pubchem.ncbi.nlm.nih.gov/ (accessed on 15 June 2023)) in the SDF file format [52]. The file format was converted to the PDB file format for molecular docking using the Open Babel GUI (ver 2.4.1).

Using AutoDock tools (ver 1.5.6), we modeled *E. signifer*’s EsigPBP3 and its binding with small molecules of eucalyptus tree volatiles through molecular docking [53]. The main process is as follows: firstly, the AutoDock software was used to preprocess the ligand’s small molecules, and the file was saved in the PDBQT format. Then, the protein model and compound ligand of EsigPBP3 were processed through the Grid module in the AutoDock software, and the parameters of the Grid Box of receptors and ligands were set (the center of the active site of the EisgPBP3 protein was set in the center of the box, and the coordinates were set as x = 7.394, y = −5.134. Z = 15.806. We set the grid spacing to 0.375 A and the grid parameter to 92 × 88 × 92, and we output the file in the GPF format. Finally, the AutoDock software was used to dock the receptor and ligand, and the conformational results of each docking were analyzed and evaluated. The results of molecular docking were analyzed, and the binding energy between the protein model and the ligands was consistently negative, with smaller values indicating stronger binding capabilities of the protein. The optimal binding site and conformation were selected based on the minimum binding energy, and their interactions were further analyzed using the Discovery Studio software 2019 (DS, Accelrys Inc., San Diego, CA, USA) and PLIP 2021 (https://plip-tool.biotec.tu-dresden.de/ (accessed on 15 July 2023)) [54]. Finally, the docking results of EsigPBP3 with sex pheromones and plant volatiles were visualized in PyMol (ver 2.5.1) [55].

## 5. Conclusions

The FCBA results of EsigPBP3 show that it has a high affinity for seven GC-EAD active ligands. The binding energy results of EsigPBP3 and the ligands show that it has the strongest ability to bind to insect sex pheromone components, followed by key volatile compounds in eucalyptus leaves, *α*-phellandrene, (−)-*β*-pinene, and eucalyptol. The key binding sites for EsigPBP3 in recognizing pheromones and volatile compounds are PHE35, MET7, VAL10, PHE38, ILE52, and PHE118. These results indicate that EsigPBP3 is one of the important PBPs in the adult *E. signifer*. The recognition of pheromones by EsigPBP3 helps the *E. signifer* adults to mate and reproduce, while the identification of volatile compounds from eucalyptus leaves also affects the oviposition choice of the *E. signifer*. This study further explores the binding characteristics of EsigPBP3 and its role in the olfactory recognition of *E. signifer* adults, searching for key ligands and binding sites as critical molecular targets for controlling the *E. signifer*.

## Figures and Tables

**Table 1:** Affinity analysis of EsigPBP3 and ligands.

EsigPBP3		
Ligands	IC_50_ (µM)	K_i_ (µM)
camphene	9.846	6.611
benzene, 1,2-diethyl-	7.192	4.829
eucalyptol	-	-
(−)-*α*-pinene	7.852	5.272
(−)-*β*-pinene	4.767	3.200
*α*-phellandrene	5.316	3.569
n-butyl ether	10.402	6.984
4-ethylacetophenone	8.261	5.547
2-phenyl-2-propanol	-	-
d-limonene	46.549	31.253
butyl acrylate	42.489	28.526
1,3,5-trimethyl-benzen	-	-

“-” means the maximum binding rate is less than 50%, and the IC_50_ (µM) and K_i_ (µM) cannot be calculated.

**Table 2:** Molecular docking energy of the compounds with EsigPBP3.

No.	Compound Name	CAS Number	Binding Energy
1	camphene	79-92-5	−5.16
2	eucalyptol	470-82-6	−5.37
3	*o*-cymene	527-84-4	−4.87
4	(−)-*α*-pinene	7785-26-4	−5.16
5	(−)-*β*-pinene	18172-67-3	−5.21
6	*α*-phellandrene	99-83-2	−5.29
7	(+)-limonene	5989-27-5	−5.0
8	2-phenyl-2-propanol	617-94-7	−5.03
9	1,2-diethylbenzene	135-01-3	−4.56
10	dibutyl ether	142-96-1	−3.14
11	butyl acrylate	141-32-2	−4.39
12	mesitylene	108-67-8	−4.65
13	naphthalene	91-20-3	−4.82
14	diacetone alcohol	123-42-2	−4.83
15	ethylbenzene	100-41-4	−4.12
16	*p*-xylene	106-42-3	−4.12
17	m-xylene	108-38-3	−4.29
18	(+)-*α*-pinene	7785-70-8	−5.2
19	myrcene	123-35-3	−4.58
20	(E)-B-ocimene	3779-61-1	−4.71
21	(E)-beta-ocimene	13877-91-3	−5.26
22	Acetophenone	98-86-2	−4.68
23	1,2-Diethylbenzene	135-01-3	−4.5
24	(3E,7E)-4,7,11-trimethyl-1,3,7,10-dodecatetraene	502-61-4	−5.98

## Data Availability

The data presented in this study are available on request from the corresponding author. The data are not publicly available due to the data in this study provide data support for future laboratory experiments.
